# Copolymers and Blends Based on 3-Hydroxybutyrate and 3-Hydroxyvalerate Units

**DOI:** 10.3390/ijms242417250

**Published:** 2023-12-08

**Authors:** Anyi Jin, Luis J. del Valle, Jordi Puiggalí

**Affiliations:** 1 Departament d’Enginyeria Química, Universitat Politècnica de Catalunya, EEBE, Av. Eduard Maristany 10-14, 08019 Barcelona, Spain; anyi.jin@upc.edu (A.J.); luis.javier.del.valle@upc.edu (L.J.d.V.); 2 Venvirotech Biotechnology S.L., Santa Perpètua de Mogoda, 08130 Barcelona, Spain; 3 Barcelona Research Center in Multiscale Science and Engineering, Universitat Politècnica de Catalunya, Campus Diagonal-Besòs, Av. Eduard Maristany 10-14, 08019 Barcelona, Spain

**Keywords:** PHBV, PHA, biopolymer blends, biocomposites, bioplastics, biomedical applications

## Abstract

This review presents a comprehensive update of the biopolymer poly(3-hydroxybutyrate-*co*-3-hydroxyvalerate) (PHBV), emphasizing its production, properties, and applications. The overall biosynthesis pathway of PHBV is explored in detail, highlighting recent advances in production techniques. The inherent physicochemical properties of PHBV, along with its degradation behavior, are discussed in detail. This review also explores various blends and composites of PHBV, demonstrating their potential for a range of applications. Finally, the versatility of PHBV-based materials in multiple sectors is examined, emphasizing their increasing importance in the field of biodegradable polymers.

## 1. Introduction: Environmental Impact of Plastic

**Figure 1:**
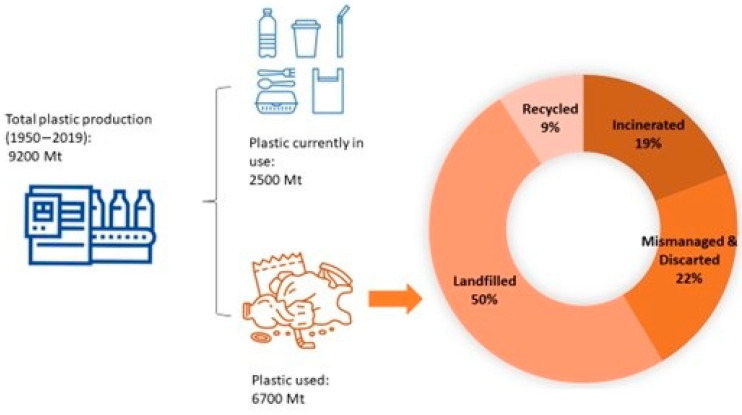
Total plastic production from 1950 to 2019 and its lifecycle. Data obtained from Ref. [1].

The exponential increase in global plastic consumption since its discovery in the 20th century has led to plastics becoming an essential part of our daily lives. Their versatility, lightweight, durability, and ease of production have established their widespread use. From the 1950s to 2019, the industry produced approximately 9200 million metric tons (Mt) of plastic (Figure 1). Currently, it is estimated that about 2500 Mt of this plastic remains in active use, representing 30% of all plastics ever produced. In contrast, the remaining 70% is classified as waste, which contributes to significant environmental contamination, as highlighted by recent studies [1].

A primary concern with plastic pollution is its high durability. Studies indicate that, depending on the material structure and environmental conditions, plastics can persist for hundreds of years [2]. For example, research by Chamas et al. [3] found that high-density polyethylene (HDPE) bottles can take around 58 years to decompose in marine environments, and HDPE pipes may persist for as long as 1200 years. This results in a significant accumulation of plastic materials on both land and sea, causing serious ecological damage. This concerning situation highlights the urgent need for sustainable, non-toxic, and biodegradable alternatives to conventional plastics [4,5,6]. In this context, bio-derived polymers like polyhydroxyalkanoates (PHAs) are gaining attention as promising and environmentally friendly alternatives [7].

### 1.1. Polyhydroxyalkanoates (PHAs): Biodegradable Alternative to Conventional Plastics

**Figure 2:**
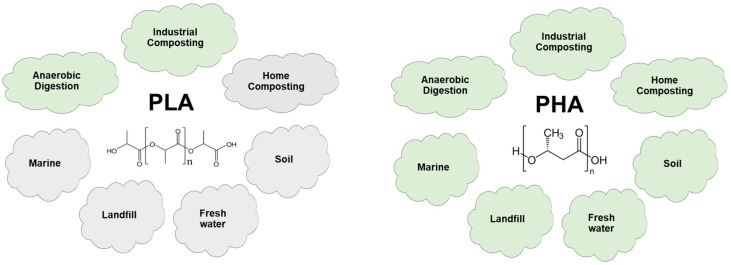
Biodegradable polymers in various environments according to established standards and certification schemes. Based from Ref. [15].

Polyhydroxyalkanoates (PHAs) are natural polyesters produced by a wide range of bacterial microorganisms [8]. These bacteria can accumulate PHAs as energy storage compounds within their cells. PHAs share similar physicochemical properties with conventional plastics such as polypropylene (PP) and polyethylene (PE) [9]. However, in terms of biodegradability, PHAs are considered 100% biodegradable materials that meet almost all established standards for biodegradation in various environments, as illustrated in Figure 2. Compared to other common biodegradable bioplastics like polylactic acid (PLA), PHAs do not require any specific conditions such as controlled temperatures or specific pH levels for complete degradation [8]. Furthermore, PHAs are among the few biopolymers known to degrade effectively not only in landfills but also in challenging marine and freshwater environments [10], where degradation is typically more complex. 

Beyond their environmental benefits, PHAs are also recognized for their biocompatibility and non-toxicity to humans [11]. These unique properties make them highly suitable for designing tissue engineering applications. In fact, a significant amount of research on PHA applications has been focused on the biomedical field, especially in regenerative engineering applications like bone regeneration [12,13,14]. Such research underscores the potential of PHAs to revolutionize both environmental sustainability and healthcare technologies. 

**Figure 3:**
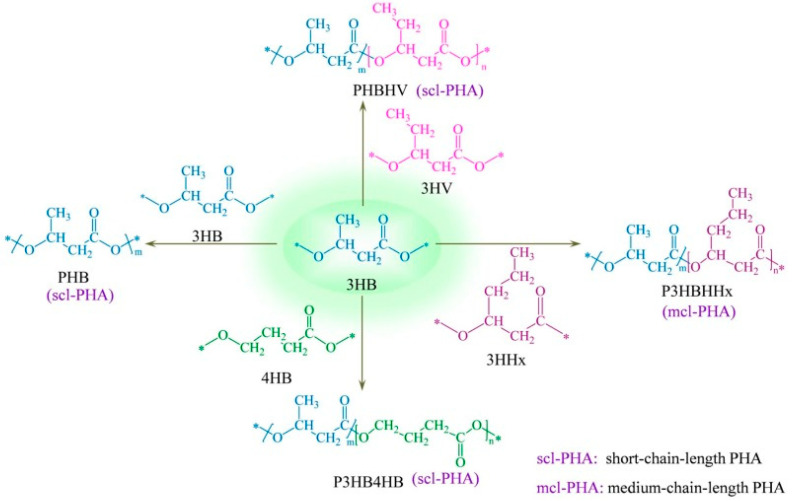
General chemical structure of PHAs. Reprinted with permission from Ref. [23]. 2023, MDPI.

To date, more than 150 different monomers have been identified within the PHA family [16,17]. Based on the length of their carbon side chains, PHAs are systematically classified into short chain lengths (4 to 5 carbons) and medium chain lengths (6 to 12 carbons), as illustrated in Figure 3. Some studies have observed the presence of long-chain PHAs containing more than 14 carbon units; however, research on these PHAs is still limited [18,19]. In addition to simple PHAs, copolymers consisting of two different monomers are also identified. Examples include poly(3-hydroxybutyrate-*co*-3-hydroxyvalerate) (PHBV), poly(3-hydroxybutyrate-*co*-4-hydroxybutyrate) (P3HB4HB), and poly(3-hydroxybutyrate-*co*-hydroxyhexanoate) (PHBHHx) [20,21,22]. These copolymers offer tunable properties, providing a variety of mechanical and thermal properties by altering the comonomer composition. 

### 1.2. Poly(3-hydroxybutyrate-co-3-hydroxyvalerate) (PHBV)

Among the various biopolymers derived from the PHA family, the copolymer poly(3-hydroxybutyrate-*co*-3-hydroxyvalerate), commonly known as PHBV, stands out for its inherent versatility. Unlike many other members of the PHA family that are derived from a single monomer and exhibit consistent physicochemical properties, PHBV offers the unique advantage of tunability. By adjusting the ratio of 3HB to 3HV monomers, the material properties can be tailored to produce products from flexible films to rigid molded objects [11]. This adaptability made PHBV more versatile and expanded its potential applications [24]. As a result, PHBV quickly attracted significant commercial interest. Companies began to explore their potential to produce biodegradable plastics on a commercial scale, providing an alternative material to traditional plastics. 

The objective of this review is to provide a comprehensive insight into PHBV, covering its biosynthesis pathways, physicochemical properties, blend compatibilities, and potential applications. 

## 2. Production of PHBV

**Figure 4:**
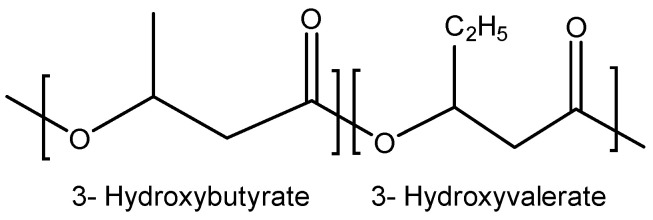
Molecular structure of poly(3-hydroxybutyrate-*co*-3-hydroxyvalerate) (PHBV).

During the 1970s and 1980s, numerous PHAs were identified [17]. Researchers discovered that certain bacteria, such as *Ralstonia eutropha* (previously known as *Alcaligenes eutrophus*), when fed a combination of glucose and propionate, produced not only hydroxybutyrate units but also 3-hydroxyvalerale (3HV). This led to the creation of the copolymer PHBV (Figure 4), which exhibited superior attributes compared to PHB, including enhanced flexibility [25].

### 2.1. Biosynthesis of PHBV

**Figure 5:**
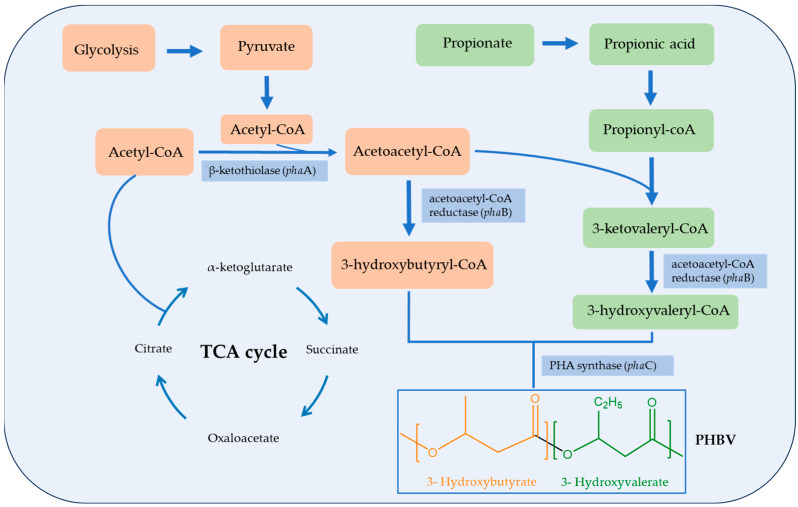
General PHBV biosynthesis pathway. Based from Refs. [24,25].

The process of PHBV production in bacteria, as shown in Figure 5, involves two main pathways. These pathways lead to the formation of two different types of monomers: a C4 monomer (3-hydroxybutyrate) and a C5 monomer (3-hydroxyvalerate) [26]. The PHBV biosynthesis process starts with the bacteria consuming carbon sources such as glucose from their environment. They break down the glucose through common metabolic processes, like glycolysis and the TCA (tricarboxylic acid) cycle, leading to the production of key intermediates such as acetyl-CoA [18]. This acetyl-CoA acts as the precursor molecule for PHBV synthesis. 

In the first key step, an enzyme called β-ketothiolase (*pha*A) condenses two acetyl-CoA molecules to form acetoacetyl-CoA. Then, another enzyme of NADPH-dependent acetoacetyl-CoA reductase (*pha*B) converts this into 3-hydroxybutyryl-CoA. At the same time, if precursors for 3HV, such as propionic acid or related compounds, are present in the medium, they can be metabolized to yield propionyl-CoA. This propionyl-CoA then combines with acetyl-CoA to produce 3-ketovaleryl-CoA, which is reduced by *pha*B to 3-hydroxyvaleryl-CoA. The final step in the pathway is the creation of the PHBV copolymer. This happens when an enzyme called PHA synthase (*pha*C) catalyzes the polymerization of previously formed 3-hydroxybutyryl-CoA and 3-hydroxyvaleryl-CoA [27]. The ratio of 3HV in the copolymer depends on the availability of precursors in the growth medium [28]. Once synthesized, PHBV is stored inside the bacterial cells as granules, which serve as energy reserves. Under nutrient-rich conditions, the bacteria can utilize them through depolymerization and metabolize the resulting monomers for energy. 

It is important to understand that while these general principles are applicable to most PHBV-producing bacteria, the specific details of PHBV biosynthesis can vary significantly depending on the bacterial strain. 

#### 2.1.1. Microbial Producers of PHBV

Several bacteria can produce PHBV naturally under certain conditions. Table 1 provides a list of various bacteria that are native producers of PHBV. 

The HV content in PHBV can significantly vary depending on the microorganism and the substrate used. It has been observed that introducing 3HV precursors can greatly increase the hydroxyvalerate content in PHBV. For example, Loo and Sudesh [47] produced PHBV with various molar fractions of 3HV, ranging from 0 to 90 mol%, using a combination of sodium 3-hydroxybutyrate and sodium valerate as carbon sources for *Delftia acidovorans*. Sheu et al. [48] achieved similar results. They used glucose and valerate as a combined carbon source for the thermophilic bacterium *Caldimonas taiwanensis*. By adjusting valerate concentrations, they were able to produce PHBV with a molar fraction of 3HV ranging from 10 mol% to 95 mol%. This capability to customize the copolymer composition is crucial for achieving specific physicochemical properties required for various applications. It also underscores the significance of selecting appropriate microbial species and substrates to obtain the targeted PHBV composition. 

Although adding 3HV precursor can effectively produce PHBV, its high cost often limits its use. Over the past decade, extensive research has focused on employing genetically engineered strains and metabolic engineering techniques to enhance PHBV yield, alter its composition, and reduce production costs. 

These genetically modified strains may be derived from the native PHBV producers (as listed in Table 1) or from other bacterial species that do not naturally synthesize PHBV. For example, Yang et al. [49] successfully engineered *Escherichia coli* to produce PHBV from glucose. They employed the citramalate pathway to generate 2-ketobutyrate, a precursor for propionyl-CoA. By optimizing metabolic pathways and gene expression, the researchers achieved a polymer content of 61.7 wt% with the 3HV monomer fraction reaching up to 5.5 mol%. The research demonstrates the potential of genetic engineering to produce PHBV from simple carbon sources such as glucose.

Since each bacterial strain has unique metabolic pathways, tailoring metabolic engineering techniques to each strain is crucial for efficient PHBV production. In recent advancements, Yong Chen et al. [50] altered metabolic pathways to regulate flux and enhance PHBV synthesis. By strategically deleting or overexpressing certain genes, they improved production pathways, increasing the target product yield. Their approach also involved using Next Generation Industrial Biotechnology (NGIB) based on extremophiles, a promising technique for continuous and open fermentation processes designed specifically for PHBV production. Furthermore, by manipulating the TCA cycle, they successfully directed metabolic flux to produce the essential building blocks for PHBV synthesis. 

Moreover, Yoon et al. [51] combined genetic and metabolic engineering to optimize *Methylorubrum extorquens* (a type of Gram-negative bacterium) for PHBV production using formate as the main carbon source. By deleting the native *pha*C gene and introducing pathway genes from other organisms, they successfully converted propionyl-CoA to 3-hydroxyvaleryl-CoA. These strategic alterations in the bacterial genome resulted in a significant increase in the production of PHBV with a desirable 3HV content. 

These advancements not only demonstrate the technical feasibility but also highlight the potential for customizing the biosynthesis pathway of PHBV production. Achieving optimal production levels and precise control over PHBV properties is crucial for successful commercialization. Future research should focus on enhancing PHBV yield and the ability to tailor its composition. This involves selecting the appropriate strain and substrate, along with developing specific genetic and metabolic engineering techniques for each unique combination. The aim is to produce PHBV with the desired characteristics more efficiently and cost-effectively.

#### 2.1.2. Mixed Microbial Cultures (MMCs) in PHBV Production

The use of mixed microbial cultures (MMCs) in PHA production has gained increasing attention due to its potential advantages and alignment with sustainable production goals. The use of MMC facilitates the handling of complex substrates and the production of PHAs without the need for sterile conditions. This approach optimizes the capital and operational costs associated with PHA production [52].

Mixed microbial cultures are constituted by various microbial species, which enable a synergistic biotransformation of multiple substrates into PHA. This means that MMC can convert various carbon sources, such as waste streams from agricultural and industrial sectors, into valuable biopolymers [53,54]. Furthermore, the inherent biodiversity of MMCs provides a more stable and resilient bioproduction process, as the microbial diversity within the culture can adapt to various substrate conditions. Additionally, the presence of multiple microbial strains allows the system to equilibrate under different stress conditions, ensuring a more consistent production process and potentially reducing the risk associated with single-strain cultures [55].

One of the pronounced advantages of MMCs in PHBV production is the efficient utilization of complex substrates, such as organic fractions of municipal solid waste, wastewater, and agricultural residues [56,57,58]. Metabolic interaction between different microbes in the cultures facilitates the breakdown of complex molecules, thus expanding the range of feasible substrates for PHBV production. Furthermore, the use of waste streams as a substrate aligns with the principle of the circular economy, facilitating waste valorization. 

Despite the clear advantages, the use of MMC for PHBV production presents several challenges. A primary concern is the variability in microbial community composition. This variability can complicate the control over the properties of the final polymer and make its management more problematic [59]. Future efforts should focus on the development of advanced monitoring and control systems capable of providing real-time insights into the microbial community’s composition and dynamically adjusting environmental conditions to maintain a stable production process. Alongside this, genetic engineering might be used to develop strains with more predictable behaviors to enhance the stability of microbial communities. This could be complemented by robust process designs that are resilient to microbial composition fluctuations. Employing mathematical modeling and simulation will be crucial in predicting and managing the impacts of these variations. Additionally, conducting comprehensive metagenomic and metabolomic studies will help us to better understand the microbial interactions, guiding effective community management strategies. Finally, performing pilot studies and focusing on scaling up successful approaches will be key in translating these research findings into practical, industrial applications. It is expected that the inherent challenges of using MMCs for PHBV production will be resolved, making the process more consistent and controllable. 

#### 2.1.3. Role of Substrates

The composition of PHBV is strongly influenced by the carbon sources used during the fermentation process. The incorporation of different sources affects the ratio of the constituent monomers 3HB and 3HV, thus influencing the properties of the resulting polymer [60].

The substrates are classified as related carbon sources or unrelated carbon sources. The first consists of precursors that can be used directly in the synthesis of either 3HB or 3HV monomers, such as propionate or valerate [48]. In contrast, unrelated carbon sources, such as glucose or glycerol, are those that require a series of metabolic conversions before their carbon atoms are incorporated into PHBV [61,62].

Additionally, substrates can be fed as single or combined sources, with each approach presenting its own set of advantages and challenges for PHBV production. Using a single carbon source generally simplifies production optimization and upscaling, making the process more predictable [63,64]. On the other hand, the use of combined sources allows controlled modulation of PHBV properties by adjusting substrate types and ratios. However, this approach makes metabolic pathways and process parameters more complex to control due to greater variability in polymer composition [48,65].

Aramvash et al. [66] explored the enhancement in HV content in PHBV produced by *Cupriavidus necator*. Their study investigated the effects of various substrates (fructose, propanol, citric acid, acetic acid, propionic acid, and meat extract) on PHBV synthesis. The results indicated that a combination of fructose with propanol and meat extract exhibited better performance in PHBV production compared to propionic acid as the sole carbon source. This was the first report on the production of PHBV with an HV content of 54.1% by a wild-type *Cupriavidus necator.*

#### 2.1.4. Industrial Waste as Substrates 

**Figure 6:**
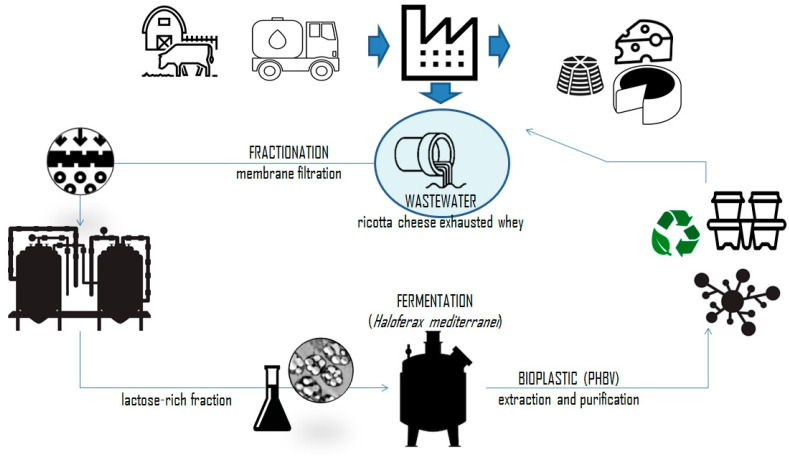
Illustration of industrial production of PHBV from Ricotta Cheese Exhausted Whey. Reprinted with permission from Ref. [68]. 2023, MDPI.

In addition to common substrates such as glucose and propionic acid, industrial waste has received increased attention as a viable substrate for PHBV production [56,64,67]. Integrating industrial waste from the agricultural, food, or other manufacturing sectors into PHBV production can effectively transform potential environmental liabilities into valuable resources. In this way, the approach not only mitigates the environmental impact of industrial waste but also represents a step towards reducing the production costs of bioplastic and therefore increasing its commercial viability. For example, Raho et al. [68] investigated the use of Ricotta Cheese Exhausted Whey (RCEW) as a substrate for producing PHBV by fermentation with *Haloferax mediterranei* (Figure 6). This substrate is a byproduct of ricotta cheese production. Fermentation conditions were optimized to maximize polymer synthesis. The resulting PHA was identified as PHBV, with a remarkably high HV content. The process was validated under pilot-scale conditions, which could be further implemented for large-scale bioplastic production, reducing the economic and environmental problems associated with RCEW disposal.

The heterogeneous nature of industrial waste can affect the composition and properties of PHBV, introducing variability that could present challenges for consistent biopolymer production. The various compounds present in waste streams can influence the metabolic pathways of the microbial strains used for PHBV synthesis, thereby affecting the copolymer composition and its physical properties. For this reason, it is crucial to develop robust microbial strains and optimize fermentation processes that can adapt to the variable nature of waste substrate while ensuring constant PHBV production [67].

### 2.2. Production Technologies

Current methods for synthesizing PHBV are associated with high production costs, making them a less competitive option compared to conventional petroleum-derived plastics. To overcome this limitation and improve the commercial viability of PHBV production, several fermentation strategies are being explored.

#### 2.2.1. Fermentation Technologies 

PHBV biosynthesis applies various fermentation techniques for microbial cultures to produce PHBV. Different fermentation strategies can significantly influence the yield, properties, and economic viability of PHBV production. Nowadays, batch fermentation, fed-batch fermentation, and continuous fermentation techniques are being applied to PHBV production. 

Among these techniques, fed-batch fermentation is the most widely used technique to produce PHBV from lab-scale to pilot-scale. It is characterized by the gradual addition of substrates during the fermentation process. By continuously supplying substrates, the microorganisms can gradually produce PHBV over a prolonged period. However, continuous fermentation is most desired for scaling up production. This technique involves a relentless process in which media are continuously added and products are constantly removed. This strategy allows for stable and continuous production of PHBV, maximizing productivity and efficiency in large-scale operations. Parroquin-Gonzalez and Winterburn [69] demonstrated the feasibility of continuous PHBV production in *Haloferax mediterranei* using volatile fatty acids. Continuous fermentations were found to be superior to batch fermentations, in which PHBV productivity normalized by cell density increased from 0.29–0.38 mg L^−1^ h^−1^ using fed-batch fermentations to 0.87–1.43 mg L^−1^ h^−1^ in continuous fermentation for carbon concentration of 0.1 and 0.25 M in the medium, respectively. 

#### 2.2.2. Downstream Processing

Downstream processing in PHBV production consists of a series of steps to recover and purify the biopolymer from microbial cells, preparing it for processing for various applications. This process is crucial as it significantly influences the overall performance, quality, and profitability of the production process [70]. 

**Figure 7:**
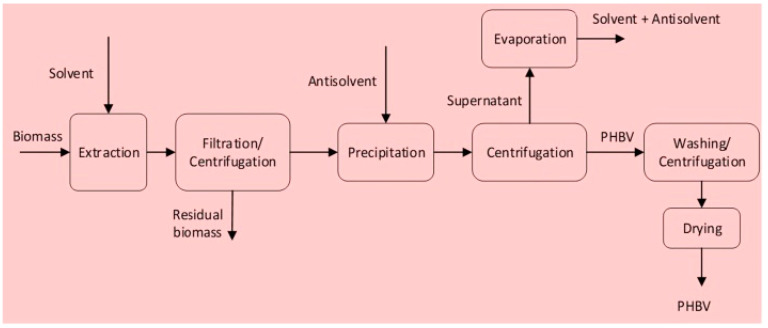
Schematic view of the extraction process to recover PHBV from biomass. Reprinted with permission from Ref. [71]. 2023, Elsevier.

Figure 7 provides a schematic view of the extraction process to recover PHBV from biomass [71]. Once the fermentation phase is completed, the microbial cells containing PHBV granules are separated from the fermentation broth using techniques such as centrifugation or filtration. The selection of a suitable cell-harvesting method depends on factors like cell size, broth characteristics, and the desired purity and yield of PHBV. After harvesting, the cell matrix is disrupted to recover PHBV. Methods such as mechanical disruption (bead milling and high-pressure homogenization), enzymatic lysis, or chemical treatment are used, each with its specific efficacy and impact on PHBV properties [72,73,74,75,76]. It is important to select a method that not only ensures optimal recovery but also maintains the integrity of the biopolymer. It is crucial to avoid the polymer degradation during this process.

The extracted PHBV often contains impurities such as residual cellular debris, proteins, and lipids. For example, one of the main problems in PHBV biosynthesis is the production of high levels of endotoxins when both Gram-negative and Gram-positive bacteria are used. The endotoxins produced can cause inflammatory, pyrogenic, and other reactions, which limits the use of PHBV in the medical sector. For this reason, the endotoxins produced must be strictly removed in the purification step [77].

Solvent extraction and precipitation are commonly used to purify the product. The choice of solvent plays a crucial role in determining the purity and molecular weight of the polymer. Solvents and conditions must be carefully chosen to preserve the properties of PHBV and align with sustainability goals. Vermeer et al. [78] explored a systematic approach to solvent screening for the extraction of PHBV from biomass. The authors constructed a database of 35 solvents and evaluated them according to specific criteria. Solvents such as acetone and dimethyl carbonate (DMC) were identified as particularly effective for the extraction of PHBV. Their research emphasized the importance of selecting the proper solvent, not only for product quality but also for its impact on the overall process design.

After purification, PHBV is dried to remove residual solvents and moisture. Techniques such as spray drying, drum drying, and lyophilization could be used [79,80,81]. In this step, it is crucial to ensure minimal thermal degradation and preserving molecular weight [82]. Finally, PHBV can be formulated or modified by blending or adding specific agents to meet specific application requirements. 

A review by Haque et al. [83] provided a comprehensive overview of the current challenges in the downstream processing of PHAs. The authors emphasized the environmental and economic problems faced in the production and processing of PHA. Despite the growing demand for sustainable and biodegradable polymers, conventional extraction methods often use toxic, expensive, and non-recyclable solvents, making the process environmentally unsustainable [83]. Given that downstream processing is a critical step in PHBV production, optimization is essential for enhancing overall production efficiency.

Future research should focus on developing more sustainable and economically viable extraction methods. This includes identifying and developing solvents that are not only effective for extracting PHBV but also environmentally friendly—exploring bio-based solvents or designing novel solvent systems to minimize toxicity and environmental impact. Additionally, developing closed-loop systems, where solvents are recycled within the process, could make the process more economically viable. This can significantly reduce both the environmental footprint and operational costs. Furthermore, integrating biotechnological advances, such as the use of enzymes or microbial systems to aid in the breakdown of PHBV granules, could facilitate easier extraction and enhance the overall process. Finally, all these methods should be translated from a laboratory scale to pilot and industrial scales, which is essential to test the scalability of new extraction methods and refine them to meet industrial requirements. 

## 3. Properties of PHBV 

PHBV is a copolymer synthesized from two different monomers: 3-hydroxybutyrate (3HB) and 3-hydroxyvalerate (3HV). The ratio of these monomers in the polymer chain can significantly affect its physical properties, particularly the mechanical and thermal characteristics. PHBV can be as crystalline as PHB or become completely amorphous by increasing the 3HV content. This variation can influence properties such as degradation rate, oxygen permeability, and other physical characteristics. The ability to adjust the 3HV ratio provides enormous versatility to PHBV, allowing it to be tailored to specific applications, whether a stiffer polymer for structural components or a more flexible one for films or fibers.

### 3.1. Thermal, Mechanical, and Rheological Properties

Yuanpeng Wang et al. [41] investigated the thermal behavior of PHBV using differential scanning calorimetry (DSC) and thermomechanical analysis (TGA) techniques, emphasizing the influence of varying HV content in the polymer. Their findings revealed that the melting temperature of PHBV decreases as the 3HV content increases, thereby expanding the processing window for molding and extrusion. The thermal degradation of PHBV occurs in a single step due to a nonradical random cleavage process, as suggested by Yun Chen et al. [82]. These observations agreed with the work of Abbasi et al. [84], who also studied the impact of 3HV content on the thermal properties of PHBV. The authors also mentioned the presence of two melting peaks in certain extracted PHBV samples, which were consistent with the results of a previous study by Guho et al. [70]. Abbasi et al. suggested that the phenomenon of two melting peaks can be attributed to isomorphism. Since PHBV has a semicrystalline structure, crystals with a higher HV content have a more pronounced amorphous phase. Consequently, these high HV crystals melt earlier during heating, leading to the first melting peak. On the other hand, crystals with a lower HV content have a higher crystallinity ratio, which causes them to melt at higher temperatures, resulting in the second melting peak. Furthermore, Abassi et al. highlighted that variations in crystalline morphologies, such as thickness, lamellar stability, and crystallite distribution, can also contribute to the formation of multiple melting peaks. 

In terms of rheological properties, the study by Abassi et al. [84] revealed that the complex viscosity of PHBV showed a reduction with the shear rate, indicating the typical shear thinning behavior of non-Newtonian fluids. At a frequency of 1 Hz, the complex viscosity increased with samples having higher HV content. 

Regarding mechanical properties, PHBV with a high 3HV content tends to have higher flexibility but low tensile strength and modulus, meaning that the material is desired for packaging applications that require high flexibility. An optimal balance between 3HB and 3HV can produce a polymer with improved toughness that combines strength and flexibility [59,84].

The ability to tailor the physicochemical, mechanical, and thermal properties of PHBV by altering the comonomer composition greatly enhances its workability. This flexibility enables PHA-producing companies to customize their products to specific client requirements. As a result, various grades of PHBV can be developed to meet diverse application needs. Since the upstream process plays a crucial role in determining PHBV composition, maintaining stability throughout this process is essential. Implementing rigorous quality control measures is indispensable to ensure the reliability and consistency of the final product before its distribution.

### 3.2. Degradation and Biodegradation of PHBV

The degradation of PHBV is highly dependent on environmental conditions. As a biodegradable biopolymer, it is explored to degrade naturally under both aerobic and anaerobic conditions. Shang et al. [85] studied the biodegradability of PHBV using lipase by placing PHBV films in a PBS medium or PBS-lipase medium. The results showed that PHBV specimens only lost about 18% of their original weight in the pure PBS medium after 7 weeks. However, in the PBS-lipase medium, weight losses of approximately 50%, 61%, and 70% were observed for PHBV containing HV fractions of 4.6%, 9.5%, and 20.7%, respectively. This feature clearly indicated that higher HV content in the PHBV structure significantly accelerated its degradation rate. The authors suggested that the enzyme molecules first degrade the amorphous regions of PHBV and then target the crystalline regions, as the degradation occurs predominantly on the surface of the polymer.

Deroiné et al. [86] explored the biodegradation of PHBV films in various marine environments. The research involved immersing PHBV films in natural seawater for a period of 180 days and monitoring the degradation process. Their findings revealed that after 180 days of immersion, the films exhibited a significant weight loss of around 36%. Scanning electron microscopy (SEM) images showed increased surface erosion and a reduction in sample thickness. 

The biodegradability of PHBV under composting conditions was studied by Weng et al. [87]. They subjected PHBV films in both pilot-scale and laboratory-scale composting environments. Their findings revealed that the degree of biodegradation of PHBV reached 81%, and complete disintegration was observed on the pilot scale. The surface morphology of the films showed signs of erosion and cavity formation, suggesting that the degradation occurred with erosion from the surface toward the interior. 

Arcos-Hernandez et al. [88] performed a comprehensive overview of biodegradation in a soil environment with PHBV film of varied HV content. They used an adjusted Hill model to estimate the time to final biodegradation of the PHBV samples. The results revealed that all of them were expected to reach 90% biodegradation between 10.7 and 22.2 months. The authors emphasized that there was no direct correlation between the copolymer composition and degradation rate. However, the polymer morphology was found to have a significant influence on the degradation mechanism, which was characterized by two main stages: biodeterioration and depolymerization. The first stage involves fragmentation of the material due to the combined action of microbial communities, and the second stage is conducted by the cleavage of polymer molecules that reduce their molecular weight. These two stages were the rate-limiting steps in the degradation process. Like other degradation processes, SEM analysis indicated that biodegradation mainly occurs on the surface of the polymer material, which started from a smooth surface and then became progressively rough, with the formation of holes, cracks, and a color change.

In summary, these studies collectively confirm the biodegradability of PHBV materials. It is important to note that PHBV with a higher HV content generally degrades more rapidly under environmental conditions than those with a lower HV content. This difference in degradation behavior underscores the importance of carefully selecting the HV ratio to suit the specific PHBV application. Furthermore, achieving an optimal balance between mechanical properties and the degradation rate of PHBV is crucial to meet the diverse requirements of various applications. For example, in the case of single-use cutlery, while optimal biodegradation is essential, the PHBV must be adjusted to a lower HV content to provide some rigidity to the material since a high HV content tends to be more flexible.

## 4. PHBV Blends

Despite advances in both the upstream and downstream PHBV production, to date, only PHBV with low HV content is commercially available. This product is manufactured by TianAn Biologic Material Co., Ltd. (Ningbo, China) under the trade name Enmat Y1000P. Several research studies on PHBV have used this material to explore various applications [89,90,91]. However, its inherent brittleness and narrow processing window due to low comonomer content limit its broader applicability. To overcome these limitations and improve their properties, strategies such as blending with other polymers and the use of composites have been investigated.

Polymer blending is a technique that involves the physical mixing of two or more polymers to create a new material with a unique set of properties that differ from the individual components. Instead of developing an entirely new polymer, which can be time-consuming and expensive, blending allows the properties of the existing polymer to be adjusted to suit specific applications or overcome certain limitations. Additionally, certain combinations can expand the processing window of PHBV, improving its manufacturing capability. Moreover, combining PHBV with more abundant or cost-effective polymers can make the resulting material more financially viable, offering a competitive advantage in markets dominated by non-biodegradable alternatives.

PHBV can be blended with a variety of polymers to achieve desired properties for specific applications. Examples include polylactic acid (PLA), poly (butylene adipate-*co*-terephthalate) (PBAT), polycaprolactone (PCL), and polybutylene succinate (PBS). 

### 4.1. PLA/PHBV Blends

PLA is a polyester widely used to blend with PHBV due to its environmental friendliness, market availability, economic competitiveness, and rapid degradation. Since PLA is more thermally stable, studies have shown that mixing PHBV with PLA can improve the thermal stability of PHBV [92]. This thermal stability is further enhanced by incorporating TiO_2_ nanoparticles. These nanoparticles not only absorb thermal energy but can also potentially catalyze the degradation process. Additionally, they help to delay the volatilization of degradation products, ensuring a balanced thermal profile for the blend. 

Mofokeng and Luyt [93] conducted further studies on the dynamic mechanical properties of PLA/PHBV blends, also utilizing TiO_2_ as a nanofiller. They revealed that the incorporation of TiO_2_ has a minimal effect on the mechanical properties of the blend. Furthermore, although the storage modulus of PLA is higher than that of PHBV in the glassy state, blending them and increasing the PHBV content tends to reduce the storage modulus of PLA. This point suggests that there is a trade-off in mechanical properties when blending these two polymers, which is crucial to consider when developing materials for specific applications where certain mechanical properties are desired. 

In a study performed by Kanda, Al-Qaradawi, and Luyt [94], morphological changes in PLA/PHBV blends were examined in detail. Their findings indicated partial miscibility between the two polymers, as evidenced by the observation of two glass transition temperatures (T*g*) for the blends. SEM studies highlighted the presence of interfacial cavities, suggesting weak interactions between the two polymers. Even though the polymers were predominantly immiscible, a “sea-island” type morphology was observed, indicating a degree of close contact between them. This observed partial miscibility could explain the mechanical property trade-offs shown by Mofokeng and Luyt [93]. 

To address the problem of immiscibility with PLA/PHBV blends, González-Ausejo et al. [95] investigated the compatibilization of PLA/PHBV blends using hexamethylene diisocyanate (HMDI), poly(hexamethylene) diisocyanate (polyHMDI), and 1,4-phenylene diisocyanate (PDI) as compatibilizing agents. Their findings revealed that the presence of these diisocyanates significantly influenced the morphology of PLA/PHBV blends. By introducing these agents, the compatibility of the polymer blend was enhanced, resulting in an increase in mechanical performance without sacrificing the thermal stability of the system. 

Regarding biodegradability, studies have been conducted on the mineralization of PLA, PHBV, and their blend in compost and soil burial environments. Muniyasamy et al. [96] investigated the biodegradation of these biobased polymers, evaluating the release of (CO_2_) over 200 days. Their findings highlighted that under composting conditions, PLA, PHBV, and their blends achieved almost 90% biodegradation. In contrast, when subjected to soil burial conditions, the biodegradation rates were noticeably lower. Specifically, the PHBV, PLA/PHBV blends, and PLA reached biodegradation levels of 35%, 32%, and 4%, respectively. The authors concluded that environmental conditions were the main factors influencing the biodegradation of these bio-based polymers.

PLA can generally be blended with PHBV without any problems in terms of processability. The partial miscibility of these materials can be further improved by incorporating various compatibilizers or nanocomposites. Additionally, the thermal stability of PHBV is improved when blended with PLA, effectively extending its processing window. Moreover, experimenting with different commercial grades of commercially available PLA to blend with PHBV could be key in attaining specific desired properties. However, it should be noted that adding PLA can significantly reduce the biodegradability of PHBV. Therefore, careful consideration is essential when using this blend in applications where biodegradation is a crucial factor. 

### 4.2. PBAT/PHBV Blends

**Figure 8:**
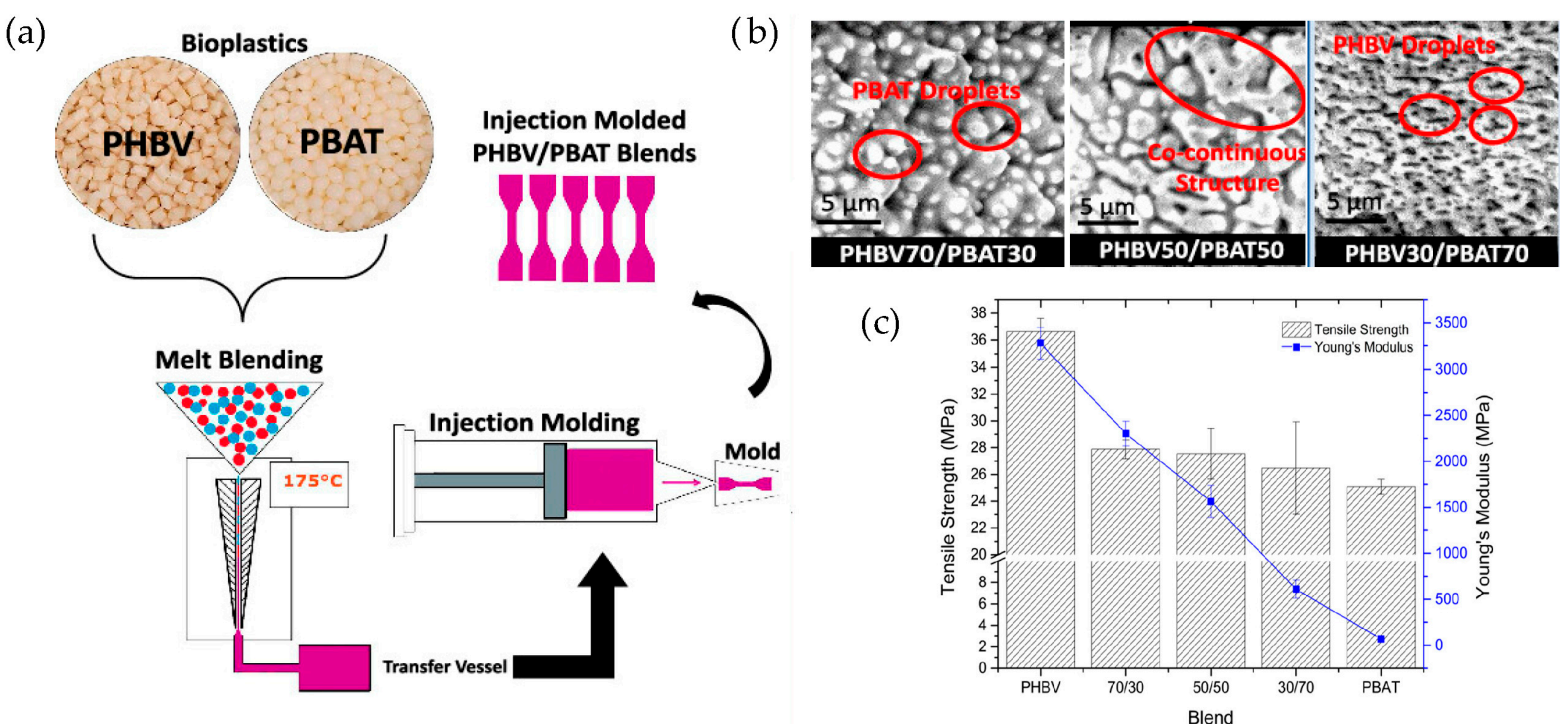
(**a**) Scheme showing PHBV/PBAT melt blend. (**b**) SEM images of the morphology of blend samples captured at 12,500× magnification. (**c**) Mechanical analysis for different weight compositions. Reprinted with permission from Ref. [100]. 2023, American Chemical Society (ACS).

Poly (butylene adipate-*co*-terephthalate) (PBAT) is a biodegradable plastic derived from petroleum sources. Researchers have explored blending PHBV with PBAT to produce fully biodegradable polymer blends suitable for a variety of applications [97]. Studies have shown that films made of PBAT can be industrially composted within seven weeks [98]. The inherent toughness property of PBAT has led to its extensive use as a blend to improve the flexibility of biopolymers, especially in packaging applications [99]. For example, Zytner et al. [100] examined various composition ratios of PHBV/PBAT blends and found that the elongation at the break of PHBV increased significantly with the addition of PBAT, as illustrated in Figure 8.

Numerous studies have indicated that PBAT and PHBV are naturally immiscible, mainly due to their lack of interaction [96,100,101,102]. However, by adjusting the composition ratios of these two polymers, properties such as melt strength, heat deflection temperature, and mechanical properties can be tailored. To improve the mechanical and thermal performance of PBAT/PHBV blends, various processing strategies and fillers have been suggested. For example, Bittmann et al. [103] explored the impact of adding functionalized montmorillonite (OMMT) to the blend. They highlighted that the addition of OMMT improved the thermal stability of PHBV. Furthermore, PBAT acted as nucleation sites, promoting the formation of additional polymer crystals. 

Pal et al. [104] proposed PBAT/PHBV-based nanocomposites to solve the problem of mixture immiscibility. They employed reactive extrusion of PHBV/PBAT to develop films suitable for packaging applications and examined the effects of organically modified nanoclays in the blend. Two distinct processing methods, compression molding and cast film extrusion, were used, and their results were compared. Initially, a master batch of nanoclay was produced by blending PBAT with 20% nanoclay using melt extrusion. This master batch was then incorporated as a reinforcement phase into the PHBV/PBAT blend matrix to create nanocomposite pellets. Films were subsequently fabricated from these pellets using both compression molding and cast film extrusion. TEM studies confirmed the effective intercalation/exfoliation of the nanoclay within the PHBV, as well as at the interface of the PHBV and PBAT blend matrix. This indicates the potential use of nanoclays to improve the overall performance of the blend, such as barrier, mechanical, and thermal properties due to their good interaction and dispersion ability. 

### 4.3. PCL/PHBV Blends 

The blend of PCL with PHBV has been extensively explored because the combination of these two materials can provide unique properties that are highly desirable in many industrial and biomedical applications. Both PCL and PHBV are recognized for their biodegradable nature, making them environmentally friendly alternatives to conventional petroleum-based polymers. PCL is known for its good mechanical properties, ease of processing, and high flexibility [105], while PHBV stands out for its biocompatibility and renewable origin. However, both have inherent drawbacks, such as the brittleness of PHBV and the slow degradation rate of PCL. The blend of PCL and PHBV aims to address these issues and improve the overall mechanical performance, processability, and biodegradability to meet the requirements of specific applications.

Qiu et al. [106] investigated the miscibility and crystallization behavior of the PCL/PHBV blend, determining that PCL and PHBV are immiscible. However, the blending process influenced the crystallization rate—specifically, the crystallization rate of PHBV decreased in the presence of PCL. Chun and Kim [107] reported similar findings, emphasizing the thermal behavior of the blends. They suggested that the nucleation of PHBV in the blend was hindered by the addition of PCL, resulting in a slower crystallization rate. The poor miscibility between PCL and PHBV is due to their low compatibility, which consequently affects the mechanical properties of blends. To address this, several studies have investigated the potential to improve compatibility using organic peroxides such as dicumyl peroxide and di-(2-tert-butyl-peroxyisopropyl)-benzene (BIB) [108,109]. In a different approach, Jenkins et al. [110] explored the production of blends in the presence of supercritical CO_2_. Their research indicated that PCL and PHBV achieve full miscibility when mixed with supercritical CO_2_.

The blend of PCL and PHBV has been processed by various methods to tailor specific properties and applications. Kalva et al. [111] employed air-jet spinning to fabricate PCL/PHBV submicron fibrous scaffolds. They evaluated the mechanical, morphological, and biological properties of the scaffolds created with different weight ratios of PHBV and PCL. Their findings indicated that scaffolds with a 50/50 PCL/PHBV ratio exhibited the best overall mechanical properties and facilitated greater cell adhesion and proliferation. The addition of PCL to the blend improved the crystallinity, thermal stability, and mechanical properties of the PCL/PHBV scaffolds. Furthermore, Kalva et al. [111] performed in vivo subcutaneous implantation studies to evaluate the compatibility of the blend with animal tissues. The results were promising with rats, as they remained generally healthy throughout the implantation period, and no significant adverse effects were observed on the implanted scaffolds. 

The Fused Deposition Modeling (FDM) technique has also been extensively used to fabricate PCL/PHBV blend scaffolds. Kosorn and Wutticharoenmongkol [112] explored various blend ratios designed specifically for cartilage scaffolds. Their findings confirmed that all scaffolds were biocompatible and showed no toxicity to cells, regardless of the mixing ratios used. The mechanical properties of the scaffolds were found to be suitable for potential cartilage applications. 

Despite the immiscibility between the PHBV and PCL, numerous advances have been made to improve the compatibility of these materials. Several applications have been explored, and the results are promising. Additional research could focus on studying the impact of blending PHBV with PCL on biodegradability and permeability barriers, which are crucial factors for applications, especially in packaging use. 

### 4.4. PBS/PHBV Blends

PBS is one of the most fascinating biodegradable biomaterials, as it is chemically synthesized from two renewable monomers, succinic acid and 1,4-butanediol. This material presents high chemical resistance, easy processability, and biodegradability. Mixing PBS with PHBV can significantly improve the thermal stability and crystallization tendency.

Phua et al. [113] investigated the mechanical and thermal properties of PBS/PHBV blends. They prepared different blend ratios of PBS and PHBV using melt blending in an internal mixer. Their findings revealed a clear phase separation in the blends, indicating poor compatibility between the two polymers. However, a moderate level of adhesion was observed between them. The DSC results showed improved crystallization behavior of PBS when blended with PHBV, but this mixing led to a decrease in the thermal stability of PBS. Furthermore, tensile test and dynamic mechanical thermal analysis confirmed the total immiscibility between both polymers. In a parallel study, Righetti et al. [114] used SEM to examine the blend surfaces. Their observations revealed a morphological transition from a dispersed phase to a continuous distribution depending on the mixing ratios. 

To address the problems of poor compatibility between PBS and PHBV, several compatibilizing agents have been explored. These include the use of organic peroxide as a free-radical grafting initiator and the incorporation of nanocomposites with halloysite clay [115,116]. In a different approach, Chikh et al. [117] used sepiolite clay together with a compatibilizing agent, which was obtained by grafting maleic anhydride onto PHBV. Their study evaluated the combined effects of sepiolite and compatibilizer by evaluating the effect of repeated extraction cycles on the morphology and functional properties of the materials. The results indicated that while PHBV is inherently more sensitive due to thermomechanical degradation, the introduction of PBS, sepiolite, and the compatibilizer provided stabilization. This led to improved morphology and maintained mechanical properties throughout multiple reprocessing cycles. The synergistic effect of both compatibilizer and sepiolite not only improved the compatibility of the blend but also improved its recyclability compared to unmodified polymers. 

### 4.5. PHBV Conjugates and Composites

Possible phase separation is one of the problems with PHBV blends when used for various applications. This issue can lead to inconsistent mechanical properties, making the material less reliable for certain applications. For example, a material that exhibits phase separation may have areas that are more brittle or less flexible than others, leading to unpredictable performance under stress. This unpredictability can be a major drawback, especially in industries where material consistency is essential, such as medical devices. To address this, significant progress has been made in the development of tailored polymeric bioconjugates and hybrid materials. Biocomposites, which combine PHBV with other materials such as natural fibers, carbon nanomaterials, nanoclays, and nanometals, have been created to improve the potential applications of PHBV [77]. A comprehensive review by Ibrahim et al. [118] described all the advances of PHBV biocomposites and their applications. 

The incorporation of nanometal or metal oxides such as TiO_2_ and ZnO can provide antibacterial performance to PHBV. Díez-Pascual and Díez-Vicente [119] studied the incorporation of ZnO nanoparticles into a PHBV matrix using a solution casting technique. Their research revealed that the resulting bionanocomposites exhibited higher thermal stability, mechanical performance, and barrier properties compared to pure PHBV. One of the most significant findings was the antimicrobial activity of the PHBV/ZnO films, which effectively inhibited the growth of human pathogen bacteria, with a notably stronger effect on *Escherichia coli* than on *Staphylococcus aureus*. The authors highlighted that the antimicrobial property, combined with the improved material characteristics, positions PHBV/ZnO bionanocomposites as promising candidates for food packaging applications, offering durability and safety. 

In a recent study conducted by Silva et al. [120], the potential of reinforcing PLA/PHBV blends with carbon nanotubes (CNTs) for electrical and electromagnetic applications was examined. The findings indicated that the CNTs served as nucleating agents for PHBV crystallization without compromising the thermal stability of the nanocomposites. Morphological images showed a uniform dispersion of PHBV domains within the PLA matrix, with CNTs predominantly dispersed in the PHBV phase. Furthermore, the results showed that the introduction of only 1.0 wt% CNTs could improve the electrical properties of the blend and provide excellent electromagnetic shielding, attenuating approximately 96.9% of the X-ray radiation.

Daitx et al. [121] have explored the effect of the organic modification of different clay minerals on the properties of PHBV nanocomposites. They specifically investigated the interactions of nanoparticles modified with (3-aminopropyl) triethoxysilane (APTES) and their subsequent impact on the final morphology and properties of the PHBV matrix. Both montmorillonite and halloysite clay minerals underwent modification to improve compatibility between the organic and inorganic phases. Cristino da Costa Reis et al. [122] further evaluated the morphology and interaction of PHBV/clay bionanocomposites for packaging applications. The barrier properties of PHBV/clay were investigated by Crétois et al. [123], using organoclay montmorillonite to improve the barrier properties of PHBV. Their study revealed that the incorporation of montmorillonite significantly improved both gas and water barrier properties. The authors stated that the best barrier effect was observed when the nanoclay structure was exfoliated, with nanoplatelets oriented perpendicular to the diffusion flow. 

In summary, PHBV alone can present several drawbacks. For example, a low HV percentage can result in high brittleness and a narrow processing window, while a high HV percentage can result in excessive flexibility for some applications. Blending PHBV with different types of polymers can help mitigate these problems. However, it is important to note that properties such as biodegradability and barrier permeability may change when blending PHBV with other polymers. The specific requirements of an application should always be considered when choosing which polymers to blend and in what proportions to ensure optimal properties. Additionally, additives such as compatibilizers and biocomposites can be incorporated to further improve the performance of the final material or to achieve specific properties, such as antibacterial or electrical characteristics. 

## 5. Applications of PHBV-Based Materials

PHBV has emerged as a promising biopolymer due to its unique combination of biodegradability and versatile material properties. In the following section, the applicability of PHBVs in different sectors is explored in detail.

### 5.1. Medical Sector

#### 5.1.1. Tissue Engineering 

Tissue engineering commonly includes tissue regeneration, which needs an environment with robust mechanical properties to facilitate the rapid growth of tissue cells. Consequently, scaffolds used for tissue engineering must be biocompatible, sterilized, and capable of maintaining mechanical integrity. Among the available biomaterials, PHA-based polymers are widely implicated due to their pronounced biocompatibility. These polymers are extensively used for bone tissue engineering but are also applied to urethral reconstructions and in the treatment of wounds using absorbable sutures [124]. 

In recent studies, Xue and colleagues [125] explored the feasibility of combining cartilage progenitor cells (CPCs) with PHBV to produce tissue-engineered cartilages. Their findings indicated that CPC-PHBV constructs were transformed into ivory-whitish, cartilage-like tissue after 6 weeks of subcutaneous implantation in nude mice. Histological examinations revealed the presence of numerous typical cartilaginous structures in the chondrocyte group and some in the CPC group, but none in the BMSC (bone marrow-derived stem cells) group. The results showed the potential of using PHBV materials in combination with CPCs to develop tissue-engineered cartilage [125].

#### 5.1.2. Drug Delivery

Drug delivery technology plays an important role in improving human health. Traditional drug delivery is limited in practical applications due to factors such as toxicity, uncertainty, and lack of selectivity. However, the use of PHA as a material for drug carriers can potentially overcome some of these limitations. PHA-based drug carriers exhibit biocompatibility and biodegradability, which significantly reduce toxicity problems associated with traditional drug delivery systems. 

The research developed by Cabaña-Brunod et al. [126] has found that PHBV nanoparticles (NPs) could be effectively used as a delivery system for iE-DAP, a NOD1 (intracellular receptor) agonist. The NPs showed controlled release of iE-DAP in-vitro, and the response of the encapsulated agonist was found to be higher than its free form. This feature highlighted that PHBV NPs can activate intracellular receptors, triggering an immune response through the release of the NOD1 agonist, iE-DAP. 

Furthermore, PHA-based drug carriers can be functionalized with various ligands for the specific treatment of different diseases. In recent studies, Alp et al. [127] developed PHBV nanoparticles for targeted delivery of etoposide drugs to osteosarcoma cells, using folic acid as a targeting ligand to exploit folate receptor-mediated endocytosis. The findings suggest that PHBV nanoparticles loaded with etoposide and functionalized with folic acid can potentially be used for the targeted treatment of osteosarcoma. 

One of the most promising advantages of PHBV in drug delivery systems is its ability to sustain drug release. This ensures that therapeutic levels are consistently maintained over a specified period, which is often a challenge in drug delivery system design. In a study by Yingjun Wang et al. [128], PHBV/HA (hydroxyapatite) composite microspheres were developed to regulate the release rate of the antibiotic gentamicin, which served as a model drug. These microspheres were created using an S/O/W (solid-in-oil-in-water) emulsion solvent evaporation method. Surprisingly, they showed an exceptionally low initial burst release of the drug. This minimal release can be attributed to the high affinity and absorbability of nano-HA particles. The sustained release of the drug lasted for more than 10 weeks. This could be particularly beneficial in applications where consistent drug release is needed over a prolonged period, such as in antibiotic therapy, to avoid frequent dosing and improve patient compliance. 

### 5.2. Food Packaging

The extensive use of conventional plastic packaging has led to critical environmental problems, including widespread plastic pollution and waste accumulation. There is a need to look for alternative materials. PHBV emerges as a promising option, as it is a bio-based and biodegradable polymer that mitigates environmental impact, ensures consumer safety, and provides effective food preservation. 

**Figure 9:**
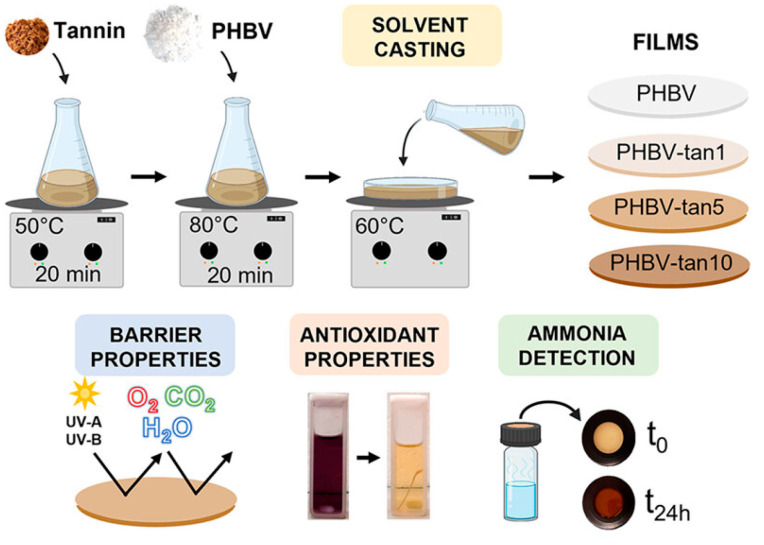
Illustration of the preparation of PHBV/tannin using the solvent casting technique. Reprinted with permission from Ref. [129]. 2023, American Chemical Society (ACS).

Ferri et al. [129] integrated PHBV with tannins to develop a fully biobased and biodegradable material suitable for food packaging. This innovative material not only reduces the environmental impact of fossil-based plastics but also introduces functionalities that extend the shelf life of food and inform consumers about its quality and freshness. The researchers prepared PHBV/tannin films using the solvent casting method, as illustrated in Figure 9. The resulting films exhibited antioxidant, UV protection, and gas barrier properties. The films were found to be suitable for temperatures ranging from refrigeration levels to those required for heating food (up to 200 °C). In terms of tensile strength, they were comparable to conventional polymers and biopolymers used in packaging. Interestingly, the PHBV/tannin films also demonstrated the ability to calorimetrically detect ammonia vapors, suggesting potential applicability as a smart indicator of food spoilage. The authors suggested that tannins could add multifunctional properties to a polymeric material, providing an attractive alternative to petroleum-based plastics for smart food packaging applications. 

Bonnenfant, Gontard, and Aouf [130] evaluated the safety and structural integrity of PHBV under different reuse conditions, including food contact, washing, and subsequent food contact. Valuable insights were provided into the potential of these biodegradable polymers for sustainable food packaging solutions. Their study revealed that PHBV exhibits an overall migration close to zero in a complete reuse cycle with certain food simulants, demonstrating its potential safety in reuse applications.

### 5.3. Agriculture

Mulch films play a pivotal role in modern agriculture, which offer numerous benefits, such as improving the overall productivity and the quality of crop production. However, traditional mulch films are often made of non-degradable plastics, which pose significant environmental challenges [131]. These films can cause soil pollution, disrupt ecosystems, and contribute to global plastic waste [132]. Tian et al. [133] provided a comprehensive review of the application of PHA in the development of biodegradable mulch films. The urgency and importance of this development was highlighted. The review analyzed the feasibility of using PHA as an alternative material and emphasized the challenges faced in its production, such as its proneness to thermal degradation during film extrusion. Various strategies, such as blending with other polymers and structural design, were explored to enhance the thermal and mechanical performance of PHA. 

Despite the potential of PHAs in the agricultural sector, a study by Liu et al. [134] highlighted significant obstacles to their widespread adoption of mulch films. These challenges include the high production cost of PHAs, their limited availability, and the need for further research to improve their mechanical and physical properties to meet the diverse demands of agricultural applications. Additionally, the authors emphasized the need to develop standards and guidelines for biodegradable mulch films to ensure their safe and effective use in agriculture. 

Intriguingly, a recent study conducted by Brown et al. [135] demonstrated that even at minimal levels of contamination, PHBV microplastics in soil could negatively affect both plant and soil health. This contamination could reduce plant growth, modify lead metabolic functions, and affect soil microbial activity and community composition. These findings reflect Liu’s concerns, emphasizing the need for caution and more research into the use of biodegradable materials in agriculture, especially when these materials can interact with soil and plants. 

### 5.4. Three-Dimensional Printing

The use of PHBV in 3D printing applications has advanced significantly in recent years, driven by the material’s biodegradability and biocompatibility. PHBV is often blended with other polymers, such as PLA, to enhance its thermal and mechanical properties, making it more suitable for 3D printing applications. Vigil Fuentes et al. [136] used styrene–acrylate copolymers as chain extenders to improve the compatibility and thermal resistance of PHBV blends. They successfully demonstrated that the incorporation of a chain extender allowed for higher printing temperature and sufficient printing speed, thus enhancing 3D printability. The optimized printed samples exhibited higher storage modulus and strength, resulting in stiffer and stronger parts. This research not only paves the way for further exploration of the use of biodegradable polymers in 3D printing but also provides a framework for developing materials that can be used in various applications, such as biomedical devices and environmentally friendly consumer products. 

The 3D-printed scaffolds of PHBV combined with calcium sulfate hemihydrate (CaSH) developed by Ye et al. [137] is another example of 3D application. They used the fused deposition modeling (FDM) technique to fabricate scaffolds and subsequently coated them with chitosan (CS) acetic acid solution. Once dried and neutralized, a chitosan hydrogel formed on the scaffold surface. Their findings indicated that resulting PHBV/CaSH/CS scaffolds significantly promoted the adhesion and proliferation of rat bone marrow stromal cells (rBMSCs). Furthermore, these scaffolds showed a higher expression level of the osteogenic gene compared to PHBV and PHBV/CaSH scaffolds, which improved the osteogenesis of rBMSCs. In vivo experiments further corroborated these results and demonstrated that PHBV/CaSH/CS scaffolds effectively promoted new bone formation. This innovative integration of 3D-printed PHBV/CaSH scaffold and CS hydrogel offers a promising approach to enhance osteogenesis properties, presenting a potential solution to repair bone defects. 

### 5.5. Textile Industry

The textile industry has been identified as a major polluter due to its dependence on petroleum-derived materials and the emission of harmful chemicals during production [138]. The use of biodegradable materials such as PHBV is gaining a lot of attention as an eco-friendly alternative. Its ability to degrade more quickly minimizes long-term environmental waste. Within the textile sector, various applications of PHBV have been explored. For example, Huang et al. [139] developed a multi-filament yarn using a PLA/PHBV blend. The resulting yarn exhibited thermal and mechanical properties suitable for standard textile and dyeing/finishing processes. Single-knit fabrics made from this PLA/PHBV filament yarn met industrial standards in terms of strength, stretch, and recovery. Furthermore, their study highlighted the excellent antibacterial performance of 100% PLA/PHBV fabrics against many microorganisms. A study conducted by Xu et al. [140] also emphasized the antibacterial properties of PHBV materials, suggesting their potential to produce hygienic textile products without the need for synthetic chemical treatments. Furthermore, the integration of reinforcing agent, such as lignocellulosic fibers, has been developed to improve mechanical limitations such as brittleness and the high cost of PHBV materials, thus expanding their applicability in the textile sector [141]. 

## 6. Conclusions

Nowadays, translating PHBV from laboratory research to real-world use still presents several challenges. Advances in genetic engineering and metabolic engineering are expected to improve efficiency and reduce the cost associated with PHBV production. Another challenge is the processability of PHBV. Its narrow processing window and susceptibility to thermal degradation present difficulties for traditional plastic-processing techniques. There is also the problem of phase separation when blending with other polymers, which can affect the overall performance of the resulting material. Achieving uniform dispersion, ensuring compatibility, and retaining desirable properties of the polymers involved are important for end applications. Overall performance, including mechanical, thermal, and degradation properties, must be optimized. 

Despite these challenges, the future of PHBV looks promising. With continued research focused on improving production yields, improving processability, and fine-tuning their properties by blending and preparing new composites, PHBV could become a more dominant player in the materials market. Furthermore, the development of innovative processing techniques and the exploration of new applications will further strengthen PHBV’s place as a sustainable material and a viable alternative to conventional plastics. 

## Figures and Tables

**Table 1:** Biosynthesis of poly(3-hydroxybutyrate-co-3-hydroxyvalerate) (PHBV) by various microorganisms and substrates.

Microorganism	Substrate	Maximum PHBV in Dry Cell (wt%)	% HV	Reference
*Azotobacter vinelandii*	Sucrose	73	35%	[29]
*Bacillus aryabhatthai*	Glucose and propionic acid	-	-	[30]
*Bacillus cereus* S10	Glucose	70	5%	[31]
*Bacillus circulans*	Dextrose	-	-	[32]
*Bacillus megaterium*	Pineapple Peel Waste	49	6–35%	[33]
	Cheese Whey Permeate	87	-	[34]
*Corynebacterium glutamicum*	Glucose	33–36	73%	[35]
*Cupriavidus necator*	Levulinic acid and sodium propionate	-	80%	[36]
*Escherichia coli*	Threonine	30	18%	[26]
*Haloferax mediterranei*	Rice-based ethanol stillage	71	15%	[37]
	Vinasse	70	12%	[38]
*Halomonas profundus*	Valerate	80–90	55%	[39]
*Halophile yangia* sp. ND199	Glycerol and yeast extract	53	3%	[40]
*Ralstonia eutropha*	Levulinic acid	81	0–41%	[41]
	Glucose	69	26%	[42]
	Madhuca indica flower extract	49	-	[43]
	Paddy straw	38	-	[44]
*Rhodococcus aetherivorans*	Toluene	18	44–66%	[45]
*Rhodospirillum rubrum*	Fructose	60–80	47%	[46]

## Data Availability

Not applicable.
